# Supplementary material for: Translation, validation and psychometric evaluation of the Persian (Farsi) version of the Low Anterior Resection Syndrome Score (LARS-P)
Source: PLoS One. 2021 Feb 25;16(2):e0247054. doi: 10.1371/journal.pone.0247054 (PMC7906462; doi:10.1371/journal.pone.0247054)
Supplement: Attachment — Submitted filename: PONE29.10.docx [file pone.0247054.s002.docx]

PONE-D-20-28626

Translation, Validation and Psychometric Evaluation of Farsi version of the Low Anterior Resection Syndrome Score

Thank you for the opportunity to review the manuscript. I reviewed this manuscript carefully with a great interest. I respectfully provided my comments below

1: It is recommended to change the title of the manuscript

**Psychometric characteristics of the Persian version of the Low Anterior Resection Syndrome Score (LARS-P)**

2: The abstract needs major editing.

3: In validity, explain face and content validity

4: In methods convergent and discriminative validity of the LARS score explain in two separate sections.

5: The standard time interval for the test re test is at least two weeks. Based on what reference did you consider this time period to be one week?

6: For test–retest reliability you must use Bland–Altman plot of the differences between LARS scores on the first and second tests.
