# Supplementary material for: Translation, validation and psychometric evaluation of the Persian (Farsi) version of the Low Anterior Resection Syndrome Score (LARS-P)
Source: PLoS One. 2021 Feb 25;16(2):e0247054. doi: 10.1371/journal.pone.0247054 (PMC7906462; doi:10.1371/journal.pone.0247054)
Supplement: Attachment — Submitted filename: response to reviewers.docx [file pone.0247054.s004.docx]

**Dear Editor in Chief of the PLOS Journal,**

**Thank you very much for considering our manuscript and for the valuable comments of the reviewers. We revised the manuscript based on the comments. All the changes are highlighted within the manuscript. Answers to the questions of the reviewers are in below.**

**Again, thanks for considering our manuscript. I really appreciate it if you may inform me if further revision is needed for our manuscript.**

**Sincerely,**

**M.R.Keramati**

**REVIEWER #1**

**Comment:** All comments have been addressed.

**Answer:** Thank you very much for your time and valuable comments.

**REVIEWER #2**

**Comment:** I recommended publication as all issues raised by the Reviewers are accounted for. I have no further comments or requirements.

**Answer:** Thank you very much for your time and valuable comments.

**Reviewer #3**

**Comment 1**: line 295 (Discussion):" Cronbach’s alpha displayed good internal consistency and reliability in our study". I believe you took this analysis out of the manuscript, hence it should not be mentioned in the discussion.

**Answer**: Thank you very much for your time and valuable comments. According to your comment, the analysis (regarding the Cronbach’s alpha) was removed from the mentioned part of the discussion section.

**Comment 2:** I recommend to show not just the minimum days between the two tests in the test-retest, but also the maximum number of days.

**Answer:** Thank you very much. In line-141 of the manuscript (Reliability part of the Methods Sections) the maximum number of days was added.
